# Analyses of Mineral Content and Heavy Metal of Honey Samples from South and East Region of Turkey by Using ICP-MS

**DOI:** 10.1155/2017/6391454

**Published:** 2017-05-11

**Authors:** Serap Kılıç Altun, Hikmet Dinç, Nilgün Paksoy, Füsun Karaçal Temamoğulları, Mehmet Savrunlu

**Affiliations:** ^1^Department of Food Hygiene, Faculty of Veterinary Medicine, Harran University, 63000 Şanlıurfa, Turkey; ^2^Department of Pharmacology and Toxicology, Faculty of Veterinary Medicine, Harran University, 63000 Şanlıurfa, Turkey; ^3^Department of Biochemistry, Faculty of Veterinary Medicine, Harran University, 63000 Şanlıurfa, Turkey; ^4^Province Control Laboratory, Ministry of Food, Agriculture, and Livestock, 63000 Şanlıurfa, Turkey

## Abstract

The substantial of mineral ingredients in honey may symbolize the existence of elements in the plants and soil of the vicinity wherein the honey was taken. The aim of this study was to detect the levels of 13 elements (Potassium (K), Sodium (Na), Calcium (Ca), Iron (Fe), Zinc (Zn), Cadmium (Cd), Copper (Cu), Manganese (Mn), Lead (Pb), Nickel (Ni), Chromium (Cr), Aluminum (Al), and Selenium (Se)) in unifloral and multifloral honey samples from south and east regions of Turkey. Survey of 71 honey samples from seven different herbal origins, picked up from the south and east region of Turkey, was carried out to determine their mineral contents during 2015-2016. The mineral contents were analyzed by inductively coupled plasma mass spectrometry (ICP-MS). The most abundant minerals were K, Na, and Ca ranging within 1.18–268 ppm, 0.57–13.1 ppm, and 0.77–4.5 ppm, respectively. Zn and Cu were the most abundant trace element while Pb, Cd, Ni, and Cr were the lowest heavy metals in the honey samples surveyed, with regard to the concentrations of heavy metals such as Zn, Cu, Pb, Cd, Ni, and Cr suggested and influence of the botanical origin of element composition. Geochemical and geographical differences are probably related to the variations of the chemical components of honey samples.

## 1. Introduction

Bees produce honey from the pollen and nectar which visit flowers, other plants, and honeydew. Honey is a kind of natural mellifluous nutrient produced from nectar and pollen grains or from secretion of living plant parts that the* Apis mellifera* bees collect [1]. Bees make honey to behave as a nutrient reservoir for the colony for hard times when the climate is adverse and there are no flowers [2].

The Codex Alimentarius [3] defines honey as follows: “Honey is the natural sweet substance, produced by honeybees from the nectar of plants or from secretions of living parts of plants, or excretions of plant-sucking insects on the living parts of plants, which the bees collect, transform by combining with specific substances of their own, deposit, dehydrate, store and leave in honeycombs to ripen and mature.”

Honey has a superior nutritional value and it is a composition of carbohydrates such as glucose, sucrose, fructose, maltose, and other polysaccharides and oligosaccharides as well as acids, flavonoids, vitamins, minerals, waxes, aroma compounds, pollen grains, pigments, and enzymes [4, 5]. The composition depends on the composition of nectar and honeydews [4]. Honey also contains a variety of macro- and microminerals that are the minor constituents of honey present in the range 0.02–1.03%. Trace elements are mainly the ash content of honey [2]. Elements are inorganic compounds which have to exist in the human body for vital activities. Elements such as Se, Cu, Mn, Fe, Ni, and Zn are essential for normal metabolism [6] but above tolerance limits they should be environmental pollutants that are hazardous for human health and trace elements such as Pb, Cd, and Al are considered as toxic and should damage the human metabolism [6, 7]. The levels of Pb, Cd, Ni, and Cr are unacceptable owing to their carcinogenic and cytotoxic influences [8]. The mineral and toxic metal content of honey have been used as a quality indicator [9]. Toxic metal levels of honey depend on the biological and geographical origin [10]. ICP-MS is a good technique with simultaneous determinations of elements, high sensitivity, capability, and wide linear range [11].

Honey has a great nutritional importance with high viscosity; also it has an antioxidant, bactericidal, and fungicidal effects [12, 13]. The physical and chemical quality of honey are influenced by its geographical origin and environmental factors [14]. Honeybees are permanently exposed to the influence of industrial pollutants through the air, soil, and water [1, 12] so some researchers highlighted the possibility of honey as a biomarker [1].

Turkey is a major producer and consumer of honey with 110.000 metric ton in the world because of favorable climatic conditions. The most widely consumed honey types in Turkey are flower honey, pine honey, chestnut honey, thyme honey, astragalus honey, and citrus honey. Turkey exports honey mostly to Germany, USA, Jordan, Hungary, Iraq, Saudi Arabia, Austria, Jordon, Hungary, Belgium, Spain, and Northern Cyprus [15].

The goal of this study was to determine the levels of 13 elements (K, Na, Ca, Cd, Pb, Fe, Mn, Cu, Ni, Cr, Zn, Al, and Se) in unifloral and multifloral samples of honey from nine provinces that have diverse geographical and environmental characteristics, mineral composition of soil, in terms of pollution, watering, capability of being absorbed by plant, fertilizer distribution, and climatic conditions of south and east regions in Turkey.

## 2. Materials and Methods

### 2.1. Sample Collection

**Figure 1:**
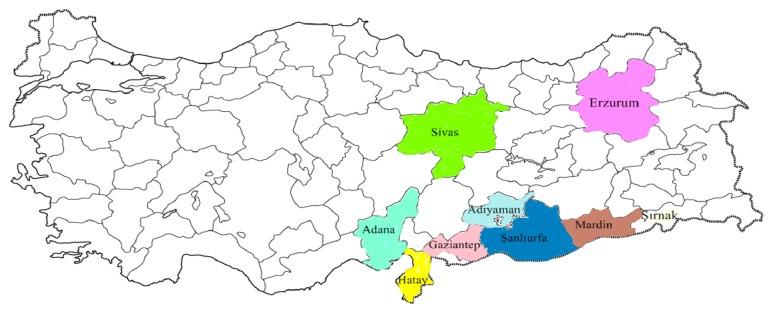
Geographical origin of honey samples.

Beekeeping is widespread in Turkey: Adana, Sivas, Şanlıurfa, Erzurum, Hatay, Şırnak, Adıyaman, Mardin, and Gaziantep. These province's honey production was in the 2015 year, respectively, 9763, 3327, 1502, 1473, 1176, 447, 418, 377, and 142 metric ton [16] (Figure 1).

Survey of 71 honey samples from nine different herbal origins, collected from the south and east region of Turkey, was carried out to assess their mineral contents during 2015 and 2016. All collected samples (ca. 200 g) were taken from the local beekeepers produced by traditional procedures with guaranteed origin and stored in closed polyethylene flasks and stored at 20-21°C in a lightless place until analysis (Table 1).

### 2.2. Reagents and Solution

Whole solutions were prepared with ultrapure water acquired by passing distilled water through a water purification system (MES MP Minipure, Turkey). All reagents were of analytical grade except otherwise stated. The standard solution of elements was obtained from standard solution Agilent Japan, Lot number: 10-160YPY2. Honey samples were digested with intense hydrogen peroxide (30% volume concentration of H_2_O_2_, Merck, Germany) and nitric acid (65% volume concentration of HNO_3_, Merck, Germany). Spike solutions were supplied from stock solutions as a multielement solution (Agilent Japan).

### 2.3. Equipment and Accessories

Elemental analysis was performed on an Agilent 7500 ce with an Octopole Reaction System Inductively Coupled Plasma Mass Spectrometer with an Auto Sampler (Cetac ASX-520) and a Nebulizer (Agilent, Japan).

Table 2 shows the instrumental parameters for ICP-MS. In the sample pretreatment (digestion procedure) a microwave oven (MARS xpress, CEM) was used.

### 2.4. Analytical Determinations

The mineral contents were analyzed by inductively coupled plasma mass spectrometry (ICP-MS) after microwave-assisted acid digestion. All glassware was cleaned with %10 (v/v) HNO_3_ solution for one day and rinsed with ultrapure water. 1.0 g of each sample was digested with 4.0 mL of 65% (v/v) HNO_3_ and 0.5 mL of 35% (v/v) H_2_O_2_ in PTFE vessels. The vessels were placed into microwave system (MARS 5, CEM). A blank digest was carried out in the same way. Digestion conditions for the microwave system applied were as follows: up to 120°C for 15 min and then constant for 10 min; up to 160°C in 20 min and constant for 15 min; finally, a cooling stage (30 min) was carried out to 22°C and diluted to 50 mL with deionized ultrapure water. This solution was finally used for elemental analysis, performed with an ICP-MS equipped with a concentric Nebulizer, a quartz torch with quartz injector tube, and cyclonic spray chamber. The concentrations of 13 elements (Na, K, Ca, Mn, Fe, Cu, Zn, Se, Al, Cr, Ni, Cd, and Pb) were determined in honey samples. The isotopes ^23^Na^+^, ^27^Al^+^, ^39^K^+^, ^44^Ca^+^, ^52^Cr^+^, ^55^Mn^+^, ^56^Fe^+^, ^60^Ni^+^, ^78^Se^+^, ^111^Cd^+^, ^208^Pb^+^, ^66^Zn^+^, and ^63^Cu^+^ were detected. All samples were analyzed in duplicate and each sample was measured in triplicate by ICP-MS detection.

### 2.5. Quality Control

Quality of this test procedure was assured by using certified reference material (NIST SRM 1515-apple). Analyses of certified reference material allowed an assessment of accuracy and precision over a range of element concentrations. Certified values indicated that observed values correlated well with certified values as shown in Table 3. The accuracy of microwave digestion method was checked by standard reference material. Three replicates were done for each sample of certified reference material and two measurements were performed for each digested sample.

## 3. Results and Discussion

Table 4 shows the concentration of 13 elements in 71 honey samples collected from the south and east region in Turkey. It is remarkable that the concentration of 13 elements summarizes wide variation and different honey samples. Among the analytes of the samples, the plenty of elements are K, Na, and Ca with average levels 45.5 ± 45.5 ppm, 2.92 ± 3.36 ppm, and 1.67 ± 0.82 ppm, respectively.

K was identified as the first of the four major inadequate* nutrients* in the Dietary Guidelines for American 2010 Advisory Committee. K benefits relevant to organic anions related to K as it arises in foods such as honey. The K levels variate over a range within 1.18-268 ppm. In accordance with our data, higher amounts of K in honey samples were previously reported in Eastern Slovakia [8], Serpentin Area in the Eastern Rhodopes Mt., Bulgaria [14], Argentine [5] and Hungarian honeys [10].

Na is an essential element for normal cell function, continuation of plasma volume, acid–base balance, and transmission of nerve impulses [17]. In the case of Na, our honey samples Na content was lower (2.92 ± 3.36 ppm) than reported from the Argentine (with a mean level of 32.2 ± 21.1 ppm) [5] and Eastern Slovakia monofloral honey samples Na (8.49 ± 1.10 ppm) [8].

Ca is an essential nutrient for bone health to ensure the calcification of the bone [18]. In this study, Ca concentrations were found to be both the lowest <1 ppm (Hatay and Şanlıurfa) and the highest 4.50 ppm (Adana) in honey samples. The Ca levels have a range of <1 ppb–4.50 ppm quite less than Eastern Slovakia [8] (20.3 ± 3.09 ppm) in monofloral honey samples and in Argentine honey samples [5] (6.92 ± 4.35 ppm). Pisani et al. [19] sample's result is predominantly rich in Ca when we compare with our study.

**Figure 2:**
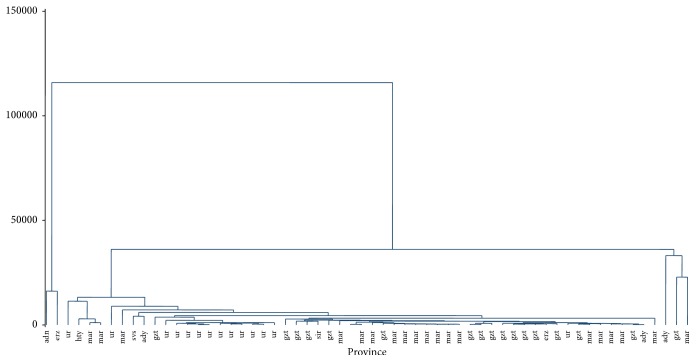
Hierarchical clustering results of honey samples (dendrogram).

Al is an abundant metal in Earth's crust. Bees are exposed to Al from many different sources when foraging for nectar externally. Our understanding of what constitutes “exposure” is likely prejudiced by a focus upon Al in the diet [20]. The highest Al concentration levels were 960 ppb in the honey samples of Adana South Anatolia which is similar to the honey samples of Çankırı province (1350 ppb). Differences between the samples may be contamination by the equipment of processing like Al containers or extractors used in the honey process [21]. The Al values of this study were lower when compared to other studies such as Anatolian honey samples of Yücel ve Sultanoğlu [22] (2540–11570 ppb). Di Bella et al. [23] and Czipa et al. [24] samples show a higher Al concentration than our results. Southeast region of Turkey honey samples have lower mean Al concentrations when compared to other studies of the world [8, 25] (Figure 2).

Se is a trace element that inherently exists in many foods such as honey. It is nutritionally essential for humans and is a component of more than twenty selenoproteins that play a critical part in reproduction, DNA synthesis, thyroid hormone metabolism, and protection from oxidative insult and infection [26]. The highest Se concentration was 65.9 ppb in the sample of Hatay province while our data is similar to other Anatolian honey samples [11, 21]. For Se concentrations determined in this study, the mean concentration was very high compared to Argentine honey samples with the mean of 10 ppb [5]. Se has an important role in antioxidant metabolism.

Fe is an essential element for the production of red blood cells. It has an ability to mediate electron transfer in the catalysis of enzymatic reactions which is also potentially toxic because it can catalyze the conversion of hydrogen peroxide into free radicals [27]. The mean level of Fe in honey samples is 269 ± 1036 ppb with the range of <1 ppb–7255 ppb. The previously detected Fe concentrations in honey samples from Southeastern Anatolia region were 2840–6660 ppb [28]. The highest level of Fe concentrations in our study is higher than the honey samples of Kahramanmaraş city with the range of 40–1210 ppb [28]. We think that the reason for this is the plant flora that grows in that region and the highest level of Argentine's honey samples of Fe concentrations 4500 ppb [5]. The Fe concentrations of the study were higher than the Romanian honeys (22.7 ppb); it can be caused by different soil and therefore vegetation diversity [1]. The Fe concentrations were a low range with honey samples from Canari Island (400–52510 ppb) [29]. Fe is an effective trace element whose deficiencies cause anemia.

Zn is generally considered to be an antioxidant and is found in nearly 100 specific enzymes [30]. It is “commonly the second most abundant transition metal in organisms” after Fe and it is the unique metal which arises in all enzyme tribes [31]. The lowest and the highest Zn concentrations were <1 ppb in honey samples from Şanlıurfa and Mardin and 237 ppb in honey sample from Hatay, respectively. Average values for Zn were slightly lower than that analyst in the literature of other Anatolia honey samples with a mean level of 1100–12700 ppb [21] and Eastern Slovakia honey samples with a range of 159–1303 ppb [8].

Cu is essential in the aerobic respiration of all eukaryotes [32]. The highest Cu content was 930 ppb in Gaziantep (South Anatolia) honey, while the mean concentration of Cu of honey samples was 68.5 ± 193 ppb. Conversely, the mean Cu concentration of our results was also high compared to other Anatolian honey samples from Kahramanmaraş province (10 ppb) [33] and low compared to Çankırı city (170 ppb) [11]. Cu levels were lower than other analysts for honey samples collected in Argentine [5].

The content of Mn in honey related to Genus* Rubus* accumulation which gives honey a typical smell [8, 34]. The maximum and minimum Mn values observed 274 ppb and <1 ppb in honey samples from Adana (South Anatolia) and Mardin (South East Anatolia), respectively. Mean values (45.6 ± 1.83 ppb) for Mn were slightly lower than those reported in the research literature for the Argentine honey samples (700 ppb) [5] whereas mean Mn levels of this study were higher than the Irish honey samples (40 ppb) [35].

The quite low concentrations of Cr, Ni, Cd, and Pb (<1 ppb) are attributed to the uncontaminated environment. In the south and east region of Turkey, the main sources of livelihood are animal breeding and agriculture so this area is not heavily industrialized. Mean Cr concentrations in Turkey were 2.4–37.9 ppb [21], 81.2–95.8 ppb [11], and 100–540 ppb [22]. Cr was more accumulated in honeydew of Slovakia honey with the range of 26.8–43.3 ppb [8].

The Ni concentrations of our honey samples are similar to the Ni concentrations of Sakarya and İstanbul provinces (013–0.88 ppb) [6]. Honey samples of Hatay city in South Anatolia Ni concentrations are within 0.13–0.4 ppb [22]. In Argentine honey samples the mean Ni concentration was 0.03 ppm. This mean data is higher than our Ni concentration [5]. Cd is an element whose source is soil passed to plants and nectar. Industrial pollution might contaminate soil or air [8]. The metals Cd and Pb are considered bioindicators for honey contamination [36]. Pb contamination generally correlates with air pollution by industry and exhaust gasses [8]. The samples of this study are collected from beekeepers who are located in mountain pasture far from the road and the factories so our data approved that.

The data of this study has shown that the honey which is produced in the east and south of Turkey does not pose a risk for heavy metals.

## 4. Conclusions

Seventy-one honey samples collected from south and east regions of Turkey were analyzed according to their metal contents. The concentrations of thirteen elements (Na, K, Ca, Mn, Fe, Cu, Zn, Se, Al, Cr, Ni, Cd, and Pb) were analyzed by ICP-MS.

The data suggested that honey samples of south and east regions of Turkey indicate the products' high quality because the concentrations of heavy metals were below the limit of detection (LOD). Also, botanical spectrum has an impact in apiculture. For more research of trace elements and heavy metal contents in honey, special attention might be demonstrated on the specific factor of honey production.

## Figures and Tables

**Table 1:** Botanical origin of honey samples.

Honey type	Number of samples	Province	Total
Unifloral	1	Şanlıurfa	20
Multifloral	19		
Unifloral	1	Erzurum	2
Multifloral	1		
Multifloral	23	Mardin	23
Unifloral	1	Adıyaman	1
Multifloral	1	Sivas	1
Unifloral	1	Adana	1
Unifloral	1	Hatay	2
Multifloral	1		
Multifloral	1	Şırnak	1
Unifloral	1	Gaziantep	20
Multifloral	19		

**Table 2:** ICP-MS operating conditions.

RF power (W)	1500
Plasma gas flow rate (L min^−1^)	15
Auxiliary gas flow rate (L min^−1^)	1
Carrier gas flow rate (L min^−1^)	1.1
Spray chamber *T* (°C)	2
Sample depth (mm)	8.6
Sample introduction flow rate (mL min^−1^)	1
Nebuliser pump (rps)	0.1
Extract lens (V)	1.5
Number of replicates	3

**Table 3:** Minerals and heavy metals concentrations in certified reference material (NIST SRM 1515-apple).

Element	Certified data (ppm)	Our data (ppm)	Recovery (%)
Na	10.0	9.94	99.4
Al	10.0	9.98	99.8
K	10.0	9.94	99.4
Ca	10.0	10.1	101
Cr	10.0	9.93	99.3
Mn	10.0	9.96	99.6
Fe	10.0	10.1	101
Ni	10.0	9.94	99.4
Cu	10.0	10.0	100
Zn	10.0	9.98	99.8
Se	10.0	9.93	99.3
Cd	10.0	10.3	103
Pb	10.0	10.2	102

**Table 4:** Elemental concentrations of honey samples.

Element	Minimum	Maximum	Mean ± SD
Na (ppm)	0.48	13.1	2.92 ± 3.36
Al (ppb)	<1 ppb	960	69.7 ± 141
K (ppm)	1.18	268	45.5 ± 45.5
Ca (ppm)	<1 ppb	4.5	1.67 ± 0.82
Cr (ppb)	<1 ppb	<1 ppb	<1 ppb
Mn (ppb)	<1 ppb	274	45.6 ± 61.8
Fe (ppb)	<1 ppb	7254.62	268 ± 1036
Ni (ppb)	<1 ppb	<1 ppb	<1 ppb
Cu (ppb)	<1 ppb	929	68.5 ± 193
Zn (ppb)	<1 ppb	237	49.9 ± 49.7
Se (ppb)	<1 ppb	65.9	54.1 ± 11
Cd (ppb)	<1 ppb	<1 ppb	<1 ppb
Pb (ppb)	<1 ppb	<1 ppb	<1 ppb
